# Haptic communication and interpersonal dynamics in hand-by-hand guided locomotion in children and adults

**DOI:** 10.3389/fbioe.2025.1622083

**Published:** 2025-08-11

**Authors:** Priscilla Avaltroni, Francesca Sylos-Labini, Margherita Villani, Germana Cappellini, Francesco Lacquaniti, Yury Ivanenko

**Affiliations:** 1 Laboratory of Neuromotor Physiology, Istituto di Ricovero e Cura a Carattere Scientifico Fondazione Santa Lucia, Rome, Italy; 2 Department of Systems Medicine and Center of Space Biomedicine, University of Rome Tor Vergata, Rome, Italy

**Keywords:** two-dimensional interactive locomotion, children, haptic interaction forces, EMG activity, interpersonal coordination, human gait

## Abstract

**Aim:**

Understanding how haptic interaction supports interpersonal coordination during locomotion is important to develop assistive technologies when necessary. While significant work has been done on haptic interactions during adult locomotion, little is known about how children interact between each other or with an adult during walking. Here, we studied haptic-guided locomotion in children and adults.

**Methods:**

We examined 11 pairs (adult-adult, child-child [6–8 years old], and adult-child) walking side by side with hand contact toward targets, with one participant leading and one blindfolded follower. The walking path was either straight or curved. We recorded and analysed upper limb muscle electromyography, kinematics, and haptic interaction forces.

**Results and conclusion:**

All dyads (adult-adult, child-child, adult-child) showed relatively small interaction forces (around 3 N), which presumably function primarily as communicative cues rather than as direct mechanical drivers of movement of the partner. Gait initiation involved compliant interaction in all dyads, with frequent anterior deltoid shortening reactions aiding arm elevation and movement onset, particularly prominent in adult-child pairs. During curved locomotion, small direction-specific adjustments in force (2–3 N) and arm elevation (3–4) conveyed effective haptic cues across ages. In addition, we found clear age-related features in the haptic interaction. Adults reduced upper-limb compliance when guiding children as compared with guiding another adult. However, children were systematically more compliant when interacting with adults, irrespective of their role, leader or follower. We interpret this difference as indicating that adults emphasize precise control and interaction stability, whereas children display more variable and reactive motor behaviour. The latter behaviour may reflect the need of children to learn and explore while walking in tandem. However, it may also reflect a compliance control that is different from that of adults.

**Limitations:**

The sample size and children age range were limited. Moreover, we only included female adults.

## Introduction

In bipedal animals—particularly humans—the freeing of the upper limbs has allowed them to be used for manipulation and communication, making touch a sophisticated tool for shared movement and interaction. From the earliest stages of development, interpersonal interactions involving physical contacts are crucial for the formation of coordinated behaviours (Arabin et al., 1996). For instance, cruising characterized by lateral movement while holding onto furniture for support or hand-by-hand walking with a parent constitute an important developmental activity in the acquisition of independent walking, typically emerging prior to the onset of autonomous locomotion in infants (Adolph et al., 2011). Furthermore, learning to interact is an integral part of learning to walk in children. The intrinsic capacity for physical coordination in early childhood facilitates the development of autonomous locomotion and interpersonal interaction, thereby establishing a foundational framework for more complex locomotor behaviours—such as cooperative and guided movement—that continue to evolve throughout the lifespan. Haptic feedback also allows individuals to synchronize motion, adjust gait, and maintain balance in joint locomotion tasks (Zivotofsky and Hausdorff, 2007; Nessler and Gilliland, 2009; Roerdink et al., 2017). An interest to the upper limb tasks in locomotion is also supported by an essential involvement of haptic communication in the interlimb and inter-subject coordination (Sylos-Labini et al., 2018), during combination of upper limb tasks with locomotion (Ivanenko et al., 2005), and in gait rehabilitation due to the interaction between cervical and lumbosacral spinal circuits (Kawashima et al., 2008; Stephenson et al., 2010; Dietz, 2011; Solopova et al., 2016; Thompson et al., 2017).

While walking with hand contact is a common situation that we naturally experience since infancy, little is known about how physical interaction forces and compliant locomotor behaviour vary across age groups, or how interactive locomotion adapts to the differing body dimensions of children and adults. Thus, investigating the basic principles that drive human-human haptic interaction during walking is important for understanding the sensory and neural processes underlying locomotor learning, gait rehabilitation, and interpersonal coordination across different age groups, including applications for human-robot interactions (Lanini et al., 2017; Sawers et al., 2017; Regmi et al., 2022). The majority of research on goal-directed locomotion has predominantly emphasized the role of vision, anticipatory adjustments, and the underlying principles governing bidimensional whole-body trajectory planning (Grasso et al., 1998; 1998; Hicheur et al., 2007; Pham et al., 2011; Belmonti et al., 2016). However, haptic communication in the context of guided locomotion has received limited scholarly attention, despite its functional significance and role in cooperative load transport (Fumery et al., 2019), child-rearing (Heiman et al., 2019), helping injured or elderly individuals (Oates et al., 2017), or guiding blind and visually impaired people via vibrotactile haptic feedback in haptic navigation devices towards a point of interest (Scheggi et al., 2014; Kappers et al., 2022). While a growing body of research suggests that humans benefit from the use of mediated touch in social and emotional contexts (Raisamo et al., 2022), as well as haptic communication has been extensively examined in stationary conditions between humans performing a shared tracking task or when tracking a randomly moving target with a robotic interface (Takagi et al., 2019; Ivanova et al., 2022; Cheng et al., 2025), there are no studies that investigated the age-related characteristics of interaction forces in guiding and coordinating locomotion.

To study haptic interactions during guided locomotion, here we examined different couples of healthy individuals (both adults and children between the ages of 6 and 8 years) walking side by side with hand contact toward different targets, when one partner was a leader and the other was a follower (walked with eyes closed). Children in this age group exhibit developmental differences in sensorimotor and cognitive functions, as well as in key mechanisms that underlie interpersonal coordination, such as proprioceptive accuracy (King et al., 2010; Cignetti et al., 2017), anticipatory locomotor adjustments (McFadyen et al., 2001; Belmonti et al., 2013; Croix and Korff, 2013), and the integration of haptic cues (Fanghella et al., 2021). Our goal was to compare a clearly defined developmental stage in childhood with the mature stage of adulthood, in order to identify potential differences in haptic communication at two distinct points in the lifespan. By analysing electromyographic (EMG) activity of the upper limb muscles, whole-body kinematics, haptic interaction forces and their directional changes (using the methodology previously developed, Sylos-Labini et al., 2018) during walking toward different targets, we aimed at characterizing haptic communication behaviour in children and adults, muscle responses during guided locomotion, and how different roles (leader vs. follower) influence motor behaviour. In particular, we focused on quantifying the mechanical effects of interactive forces, the expression of compliant behaviour, and the specific features of haptic interaction forces during guided walking.

## Methods

### Participants

**FIGURE 1:**
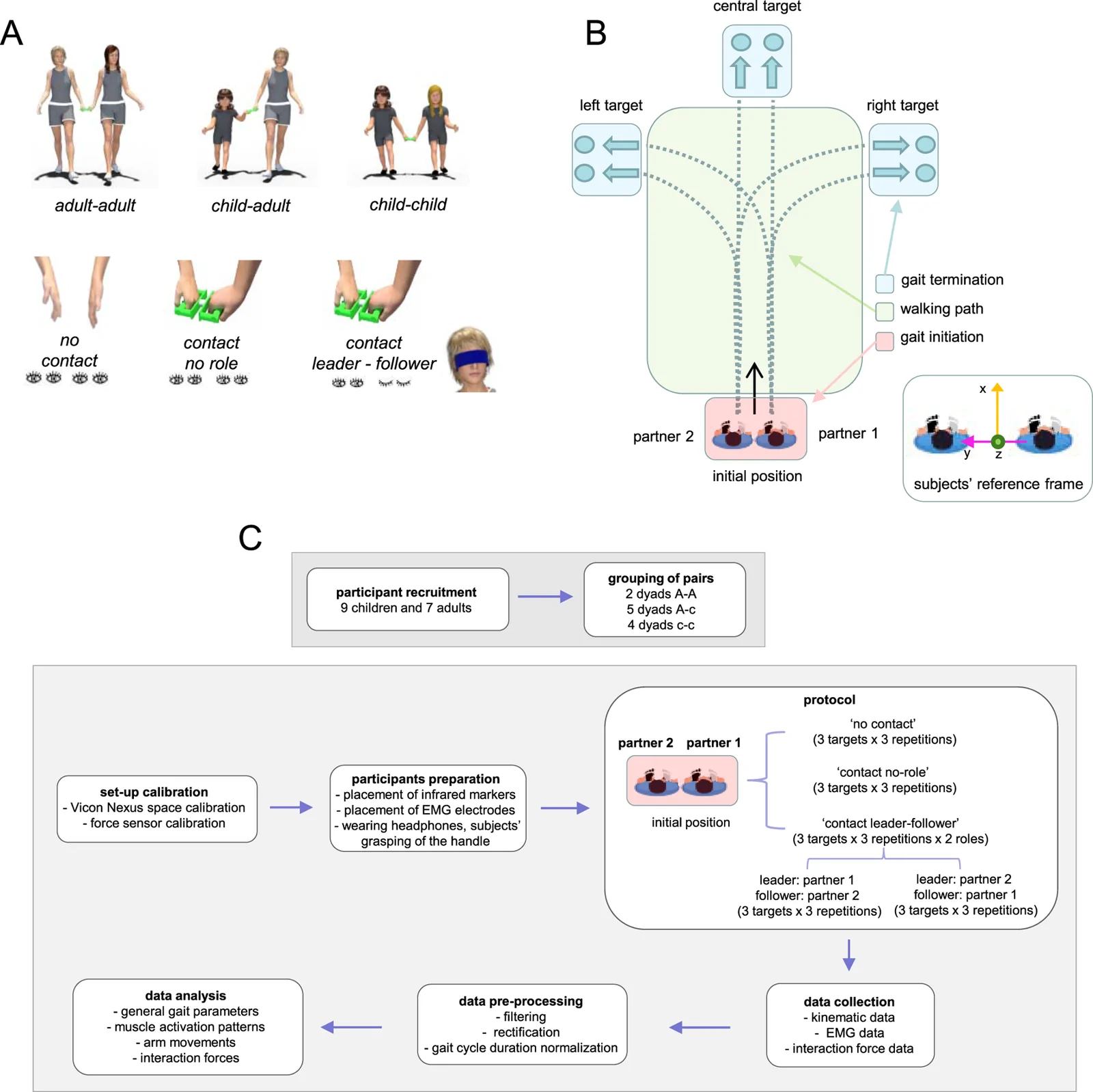
Experimental set-up and protocol. **(A)** Schematic view of participating different-sized agents (adult-adult, adult-child, child-child) and experimental conditions: ‘no contact’ (subjects walked side-by side together with eyes open and without hand contact), ‘contact no role’ (subjects walked side-by side together with eyes open and with hand contact), ‘contact leader-follower’ (subjects walked side-by side, one partner was a leader and walked with eyes open, while the other was a follower and walked with eyes closed). **(B)** The protocol was represented schematically, with the dyad walking from a starting point to three separate targets (left, central, and right). We examined three parts of the trial: gait initiation, walking (central) path, and gait termination. On the bottom, the subjects’ coordinate frame: y refers to the line that connects the two partners’ contact arm shoulders projected to the horizontal line, x - the horizontal normal to this line, and z - the vertical. **(C)** The flow diagram with the main steps of the experiment.

Nine healthy children (mean age 7 ± 1 years [mean ± SD], range 6–9 years, 3 males and 6 females, mean height 1.24 ± 0.04 m, mean weight 29.4 ± 3.5 kg) and seven healthy adults (mean age 36 ± 7 years, all females, mean height 1.72 ± 0.18 m, mean weight 60 ± 9.5 kg) participated in the study and were paired into eleven different dyads (Figure 1A): two dyads adult-adult (A-A), five dyads adult-child (A-c) and four dyads child-child (c-c). The Ethics Committee of IRCCS Santa Lucia Foundation approved the study procedures (protocol n. CE/2023_004) that adhered to the Declaration of Helsinki for medical research involving human participants. At the start of the experiments, written informed consent was obtained from all adult participants after a clear explanation of the study’s aims and procedures. For children, consent was provided by their parents, who were fully informed about the research, the procedures involved, and their right to withdraw at any time without consequences. The consent process ensured participants and their families understood the information and respected their autonomy, privacy, and wellbeing.

### Protocol

Experiments were performed in the Laboratory of Neuromotor Physiology, IRCCS Santa Lucia Foundation. The duration of the experiment was ∼1 h (including placement of EMG electrodes, infrared reflective markers, and force sensor calibration). Each dyad was instructed to walk to randomly reach three different targets (left, straight ahead, and right, Figure 1B) at ∼6 m from a starting point under three different settings (Figure 1A, bottom):

- walking without hand contact when both subjects’ eyes were open (“no contact”, the two partners walked simultaneously but independently);
- walking hand in hand through a handle connected to the force sensor while both participants’ eyes were open (“contact no role”);
- participants in each dyad walked hand in hand while switching roles: one partner was the leader, directing the follower towards the three predetermined targets (“contact leader-follower”), while the other partner was the follower, blindfolded to prevent visual input.

The initial position was always the same and the locations of the targets were marked on the ground (Figure 1B). For turning to the left or right targets, the participants were instructed to make the turn naturally approximately in the centre of the walking path (Figure 1B). For each target and each condition, we recorded three trials. Participants walked at their natural cadence, no instructions were given about the walking speed. In the first and second conditions, both partners received an audio signal at the same time to begin walking; in the third condition, only the leader wearing headphones received this signal. The follower was told to follow the leader to the target so that he/she could only use haptic communication to start and complete walking. In sum, in the first two conditions both subjects (with eyes open) knew when to start and which target to reach. In the other condition, the follower had to follow the leader blindfolded and being unaware of the precise target; only the leader had such knowledge. After reaching the target the follower was led to the starting point on a random trajectory to start the subsequent trial.

In the third condition, we recorded six trials instead of three since one partner was the leader in three of them and the follower in the other three. As a result, we recorded the following pairs in this condition: A-**A** (adult-adult), c-c (child-child), A-c (adult-child), and c-**A** (child-adult), the follower in each pair is indicated by the second bold letter, which we will use for the follower throughout the document. A total of 36 trials were recorded for each dyad, with a 2-min break in between (3 targets, 3 repetitions, and 4 circumstances [no contact, no role, leader-follower, and follower-leader]). The experiment always started with the first block of nine randomized trials in the first ‘no contact’ condition (3 targets × 3 repetitions). For the conditions in which the participants walked with hand contact (‘no role’ and ‘leader-follower’), the order of targets to reach and the conditions were selected at random. The initial left–right positioning of the two partners in the dyad was kept constant across all trials for each target and walking condition. The flow diagram of the experiment is depicted in Figure 1C.

### Data recording

Bilateral full-body kinematics was recorded at 200 Hz by means of Vicon-Nexus system (Oxford, United Kingdom) with 10 cameras placed around the walking path. Infrared reflective markers were attached on each side of the child to the skin overlying the following landmarks: shoulder joint (SHO), elbow joint (ELB), wrist (WR), third metacarpal joint (3 MC), hip joint (greater trochanter, GT), knee joint (lateral epicondyle, LE), ankle joint (lateral malleolus, LM), heel (HEEL) and fifth metatarso-phalangeal joint (5 MT).

Electromyographic (EMG) activity was recorded bilaterally by means of surface electrodes from 3 upper limb muscles simultaneously: anterior deltoid (DELTa), posterior deltoid (DELTp), medial deltoid (DELTm). EMG data were recorded with the wireless Delsys Trigno EMG system (Delsys Inc., Boston, MA), bandwidth of 20–450 Hz, overall gain of 1,000, and digitized at 1000 Hz.

The interaction forces between the two partners’ hands were recorded using an ATI Nano25 six axis force/torque sensor (Apex, North Carolina, United States) with two custom-made wood/aluminium handles attached to either side of the sensor to allow the subjects to make hand contact (Italian patent 102016000132368). Force data were digitalized at 1,000 Hz. Sampling of kinematic, EMG and force data was synchronized.

In addition, careful consideration was given to the positioning and calibration of the force sensor and the handle used for hand contact. To this end, 7 markers were placed on the handle with the force sensor in order to accurately track its position and orientation in space. These markers enabled precise motion capture and monitoring of the handle’s movements in three-dimensional space. Prior to the experimental session (‘force sensor calibration’ in Figure 1C), baseline voltage levels of all three (x,y,z) force components (while the sensor was placed on a surface and oriented horizontally) were recorded and subtracted from the collected data during the subsequent data analysis to correct for any sensor offset, thereby enhancing the accuracy of force measurements. This setup ensured that force measurements reflected precise and reliable haptic interactions during guided locomotion.

### Data analysis

**FIGURE 2:**
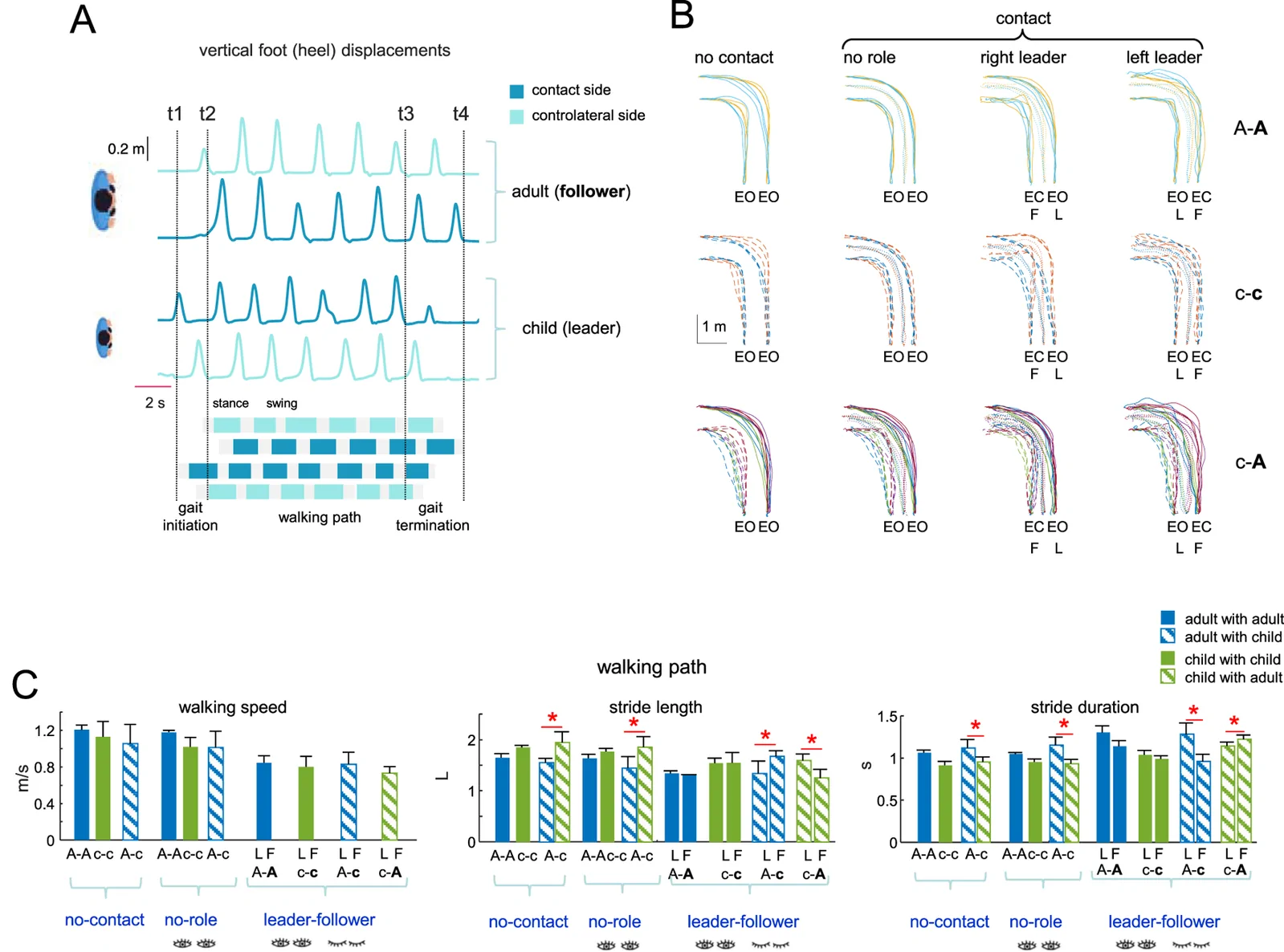
Dyad’s general performance. **(A)** Example of foot motion (vertical heel displacements and footfall patterns for a dyad A-c, adult is a leader and child a follower) and corresponding definition of three phases of the trial (gait initiation, walking path, gait termination) based on the vertical foot displacements of the leader for gait initiation and both leader and follow for gait termination: t1, onset of gait initiation; t2, onset of the walking path; t3, onset of gait termination; t4, gait termination. **(B)** Examples of superimposed trunk trajectories (displacement of the middle point between two hip markers) of different dyads **(C)** toward the left target during recorded conditions (EC, eyes-closed; EO, eyes-open). The last two columns distinguish between trials in which the leader was on the right or left relative to the follower. **(C)** General gait parameters (walking speed, stride length, stride duration) for all dyads and conditions during walking path (mean + SD). The follower in the dyad is marked by the second bold letter (A-A, c-c, A-c, and (C–A). Stride length was normalized to the limb length L (thigh + shank). Horizontal red lines and asterisks denote significant differences between subjects in the dyad (One-way ANOVA with Tukey’s HSD, p < 0.05).

For each trial, we analysed separately the three parts of the travelled distance: gait initiation, walking path and gait termination (Figure 1B). Gait initiation was defined as the time between first lift-off (t1, Figure 2A) and first heel strike of the other leg of the leader (t2, Figure 2A). Walking path was defined as the time between first heel strike and second-last heel strike. Gait termination was defined as the time between second-last heel strike of the leader (moment t3 in Figure 2A) and final heel strike of the follower (t4, Figure 2A).

### Kinematics

Kinematic data were low pass filtered at 20 Hz with a zero-lag 4^th^ order Butterworth filter. Gait cycle was defined as the time between two successive foot–floor contacts by the same leg according to the local minimum of the heel (HEEL) marker. The timing of the lift-off was determined similarly (when the 5 MT marker was elevated by more than 2 cm). General gait parameters included: walking speed, stride length, and stride duration. Walking speed for each stride was computed as the mean speed of the horizontal trunk movement, the latter being identified by the time course of the displacement of a virtual marker located at the midpoint between left and right GT markers. We reported the walking speed as the mean speed of the two participants (speed of the dyad). The stride length was defined as the distance between consecutive heel normalized to the limb length (L, determined by summing lengths of the thigh and shank segments). Bidimensional trunk trajectories in the horizontal plane from the starting position to the targets were illustrated using the displacement of the centre point between the two hip markers of the subject.

We also used the kinematic data to define the subjects’ coordinate frame and corresponding handle orientation during guided walking: y stands for the line that connects the two partners’ contact arm shoulders projected to the horizontal line, x for the horizontal normal to this line, and z for the vertical. The resulting subjects’ coordinate frame was orthogonal, as shown schematically in Figure 1B. Data were time-interpolated over the gait initiation and termination phase or over individual gait cycles during the walking path to fit a normalized 200-point time base. To characterize the contact arm behaviour of both the leader and the follower (the entire arm was modelled as the vector connecting the shoulder and wrist markers), we examined the range of motion of arm oscillations in the x direction during the walking path, and the arm elevation angle during gait initiation and termination.

### Muscle activity

The raw EMG signals were high-pass filtered at 30 Hz, full-wave rectified, and low-pass-filtered at 10 Hz with a zero-lag 4th order Butterworth. Similar to the kinematic data, also the processed EMG data were time-interpolated over a normalized 200-point time base, and averaged across participants for illustrating its general characteristics. While we analysed the three phases of the path separately, we specifically reported the results for gait initiation since in this phase we obtained the most significant effects (see Results). To describe compliant or resistive muscle behaviour during gait initiation, we deducted its minimum (baseline level) and normalized to the maximum value during the gait initiation phase. Then, we compared the mean EMG activity at the beginning (the first 30%) and end (the last 70%) of gait initiation.

### Interaction forces

Since the orientation of the handle depends both on how the participants hold it and on changes in their body orientation during 2D turning locomotion, the interaction forces were transformed and analysed within a subject-oriented reference frame that continuously updated during walking. Markers placed on the handle enabled precise motion capture, allowing us to track its movement in three-dimensional space. The three-dimensional forces were transformed from sensor to subjects’ moving coordinate system as stated above (y - direction between the two participants’ contact arms’ shoulder markers projected to the horizontal line, x - normal to y direction, and z - vertical direction). Force signs were defined relative to the partner 1 (the one positioning on the right, see Figures 1B,C): a positive x-force indicates a forward-directed pulling force exerted by the partner, a positive y-force indicates a lateral push, and a positive z-force indicates an upward (vertical) pull exerted by the partner.

Force data were low-pass filtered at 20 Hz with a zero-lag 4th order Butterworth filter. The components (x,y,z) of the contact force, the overall 3 days force, and the direction of the contact force vector during guided locomotion were all analysed. In addition, as a proxy of whole arm stiffness in adults and children during the walking path, we computed the ratio of 3 days interaction force range (difference between maximum and minimum value of the total 3 days force) to arm length (distance between shoulder and 3 MC markers) range.

In order to evaluate changes in the contact force orientation specifically associated with guided walking, we employed the previously reported approach of characterising the density distribution of the three-dimensional force vector (Sylos-Labini et al., 2018). To evaluate how the orientation of contact forces (*F*) changed during the second half of the walking path (when the subjects were expected to turn to the left or right targets, Figure 1B) compared to their orientation at gait initiation (*F*_*0*_), we computed the spherical distribution of the difference vectors (*F* − *F*_*0*_) and identified the azimuth angle associated with the region of highest density. The spherical contour of the density distribution of the 3 days force vector was calculated in Matlab adapting the algorithm proposed by Vollmer (1995) and based on the modified Kamb method (Kamb, 1959). Briefly, if *n* points are selected randomly from a uniform population distributed over an area *A*, the probability that any given point will lie within an arbitrary subarea *a* of *A* is p = *a/A*. The number of points occurring within area *a* can be considered as a binomial random variable (which mean is *μ* = *np* and standard deviation is *σ* = *pnp* · (1 − *p*)) with an expected count *E*, equal to the mean *μ*. Kamb (1959) selected a binomial probability model with *E* = *μ* = 3*σ* so that, given a random sample from a uniform population, the counting circle would be large enough so the observed counts would not be likely to fluctuate wildly from the expected count. Contour levels greater than 3*σ*(*E*) indicate a density higher than expected for a uniform distribution, and levels less than 3*σ* indicate a density lower than expected. In the algorithm, the nodes of a regular square (30 × 30) grid are back-projected onto the sphere using a stereographic projection. For each node on the sphere, the number of data points that fall within a spherical cap of area *a* = 2*π* · (1 – cos *θ*) (where *θ* is the semi-apical angle of the cap) were counted with an exponential weighting function in order to smooth the contour. For directed data distributed on a unit hemisphere of area 2*π*, the angle *θ* can be calculated considering that *p* = *a/A* = (1 − cos *θ*)/2.

The azimuth angle of the contact force vector that corresponded to the direction of the point of maximum intensity in the participants’ moving coordinate system was used to estimate the redirection of the vector during the latter half of the walking path in guided locomotion. We presented the findings from this analysis in two configurations: leader on the right - follower on the left, and leader on the left - follower on the right. This is because the directional guidance is dependent on whether the leader is on the left or right with regard to the follower.

### Statistics

Descriptive statistics included the calculation of the mean and standard deviation (SD) of the assessed variables. One-way ANOVA was used to evaluate differences between partners in the same dyad on different variables (stride length, stride duration, arm swing range of motion and arm stiffness). One-way ANOVA was also used to evaluate changes in muscle activity during gait initiation (first 30% vs. last 70% of the gait initiation interval) and to evaluate the effect of different targets (left, centre, right) for each dyad on different variables (azimuth angle of interactive forces, mean value of mediolateral angle and mean value of mediolateral force). If ANOVA resulted in a significant effect, then a Tukey HSD (Honestly Significant Difference) post-hoc test was used to detect differences between groups, conditions, targets or periods of gait initiation. Statistics on correlation coefficients was performed on the normally distributed, Z-transformed values. Statistical analysis of circular data (Watson-Williams test) was used to characterize the mean orientation of the azimuth angle of the maximum intensity of the interactive forces and its variability across steps for each dyad. Reported results are considered significant for p < 0.05.

A post-hoc power analysis was performed to assess whether the sample size was sufficient to detect the significant group difference observed in the ANOVA. The analysis was based on the effect size (Cohen’s d) calculated from the observed means and pooled standard deviation. Power estimation was conducted using a two-tailed t-test with an alpha level of 0.05. Statistical power varied across the examined variables, with values ranging approximately from 0.60 to 0.95, depending on the magnitude of the observed effect sizes. In most cases, power values exceeded the commonly accepted threshold of 0.80, indicating an adequate sensitivity to detect between-group differences. These results suggest that the sample size was generally sufficient to support the reliability of the statistical comparisons. The specific parameters analysed, along with the corresponding findings, are discussed in the following sections.

## Results

### General gait parameters and performance

Our study investigated how adults and children coordinate movement through haptic communication during side-by-side guided locomotion. Dyads were instructed to walk hand in hand toward randomly assigned targets under varying conditions (Figure 1), with participants alternating roles throughout the task (see Methods).

All dyads successfully completed all conditions to reach the targets. Figure 2A illustrated an example of footfall patterns and vertical foot displacements of two participants (c-A), served also for the definition of the three phases (gait initiation, walking path and gait termination) of the trial, while Figure 2B illustrates examples of trunk trajectories of different dyads (A-A, c-c, c-A) towards the left target under recorded conditions. In some conditions, the follower’s trajectories were more dispersed, particularly in c-c or c-A (for children or due to the lack of vision). Still, as is common for individual performance when turning towards different targets while walking (Pham et al., 2011), the trajectories were relatively smooth, with both the leader and the follower reaching the targets fairly accurately (with a precision of ∼0.2 m with respect to the location of the targets marked on the floor). When the subjects were of different heights (like in Figure 2A) their stride length and stride duration did not typically match.

The general gait parameters are shown in Figure 2C. The path taken toward the left (or right) target was not identical for the subjects on the left or right side of the dyad (the path is longer for the subject curving toward the external side). Similarly, during turning, the inner and outer legs of the subject take slightly shorter and longer strides, respectively (Courtine and Schieppati, 2003). In Figure 2C, we have illustrated the averaged parameters for stride length and duration across all turning conditions (left, central, and right targets), and for both the inner and outer legs, to highlight general trends across all conditions. The mean walking speed was comparable across all dyads in the leader-follower condition (∼0.8 m/s), although it was somewhat slower compared to the “no contact” or “no role” conditions (∼1.0 m/s, Figure 2C, left panel). In age-diverse pairs (adult-child), significant variations in stride length and duration (Tukey HSD, p < 0.05) were observed between the two partners under all conditions (no contact, no role, leader-follower) likely due to size differences.

### Gait initiation in interactive guided locomotion

**FIGURE 3:**
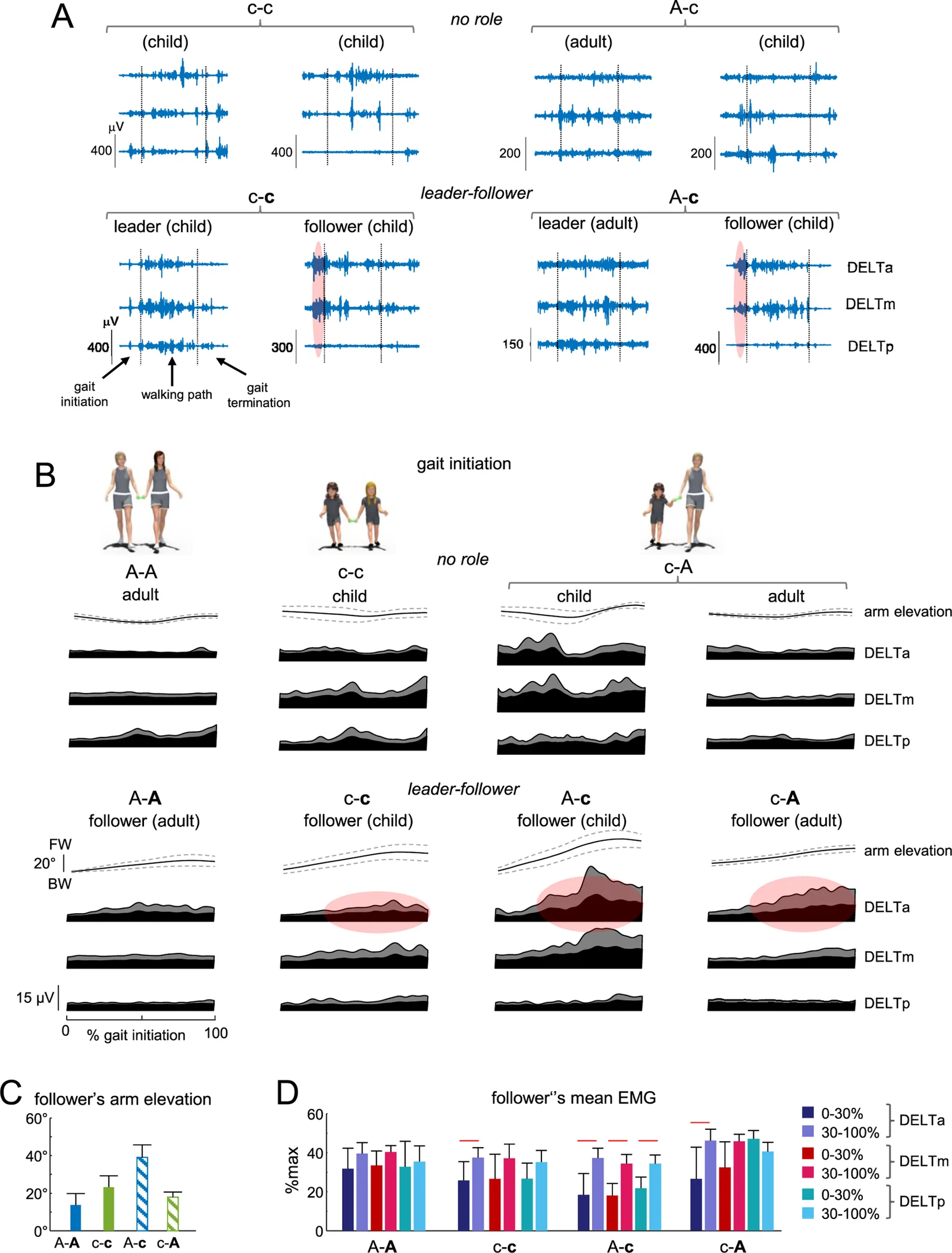
Arm muscle activity during gait initiation. **(A)** Examples of EMG activity of the contact arm muscles in the two dyads in the ‘no role’ condition (c-c and A-c, upper panels) and in the ‘leader-follower’ condition (c-c and A-c, lower panels) during walking toward the central target. Gait initiation, walking path and gait termination are separated by the vertical dotted lines. Note prominent EMG activity of DELTa and DELTm of the follower during gait initiation (marked by reddish areas in the lower panels). **(B)** Ensemble-averaged (mean ± SD) contact arm elevation angle in the sagittal plane and ensemble-averaged EMG activity (mean + SD) of shoulder (deltoid) muscles in all dyads during gait initiation. Upper panels represent the ‘no role’ condition. Lower panels show data from followers in the ‘leader-follower’ settings. The reddish spots in the lower panels schematically represent enhanced EMG activity in the course of gait initiation. FW, forward; BW, backward. Data are plotted vs. normalized gait initiation phase. **(C)** Contact arm elevation angle of the follower during gait initiation. **(D)** Mean EMG activity of follower’s shoulder muscles at the beginning (first 30%) and end (last 70%) of gait initiation, expressed as % of maximum across all trials during gait initiation. Horizontal lines denote significant differences (Tukey HSD, p < 0.05). The follower in the dyad is marked by the second bold letter (A-A, c-c, A-c, and (C–A).

Figure 3A illustrates an example of EMG activity of the deltoid muscle of the contact arm in a dyad A-c and c-c during guided locomotion (lower panels) and for comparison also in the ‘no role’ condition (upper panels). We specifically recorded the activity of proximal (shoulder) muscles since they are most active during bipedal human locomotion (Ivanenko et al., 2006; Kuhtz-Buschbeck and Jing, 2012; La Scaleia et al., 2014). We did not observe consistent responses during direction-specific smooth turning trajectories (Figure 2B) in our hand-by-hand guided walking experiments because the EMG activity was generally fairly small and rather variable across strides and participants during the walking path (Figure 3A). However, we observed frequent systematic responses in the follower’s muscles during gait initiation (highlighted by radish areas in Figure 3A).

Figures 3B–D reports the results on the follower’s upper limb movement features in different dyads during gait initiation. For comparison, we also plotted the averaged EMG patterns in the ‘no-role’ condition (Figure 3B, upper panels). In the ‘leader-follower’ conditions, a child was more compliant as a follower when walking with an adult (i.e., in the A-c dyad), since his or her arm elevation angle (in the sagittal plane) was significantly higher than for A-A, c-c and c-A dyads (Figures 3B,C). Children may have lower arm resistance due to their thinner and lighter upper limbs compared to adults, but also have a prominent (in μV, Figure 3B) muscle shortening response in the anterior deltoid, which contributes to compliant interaction during gait initiation. Despite some variability in EMG, this prominent response was observed in over 50% of trials and children. To quantify this augmented EMG activity, we calculated the mean EMG value at the beginning (the first 30%) and end (the last 70%) of gait initiation. When expressed in percent of maximum across all trials during gait initiation, EMG activity in the anterior deltoid increased significantly in c-c, A-c, and c-A dyads (Tukey HSD, p < 0.05, Figure 3D).

In the ‘no role’ condition (Figure 3B, upper panels), some muscles—such as those of the child in the c-A condition—exhibited increased baseline activity and modulation. This may be partly attributed to the child’s more relaxed arm posture during gait initiation when acting as the follower, or to the higher walking speed (Figure 2C, left panel) and differing changes in arm elevation angle observed in the ‘no role’ condition. However, we did not find significant differences in the mean EMG value at the beginning (the first 30%) and end (the last 70%) of gait initiation (p > 0.05, One-way ANOVA).

### General characteristics of haptic interaction forces in children and adults

**FIGURE 4:**
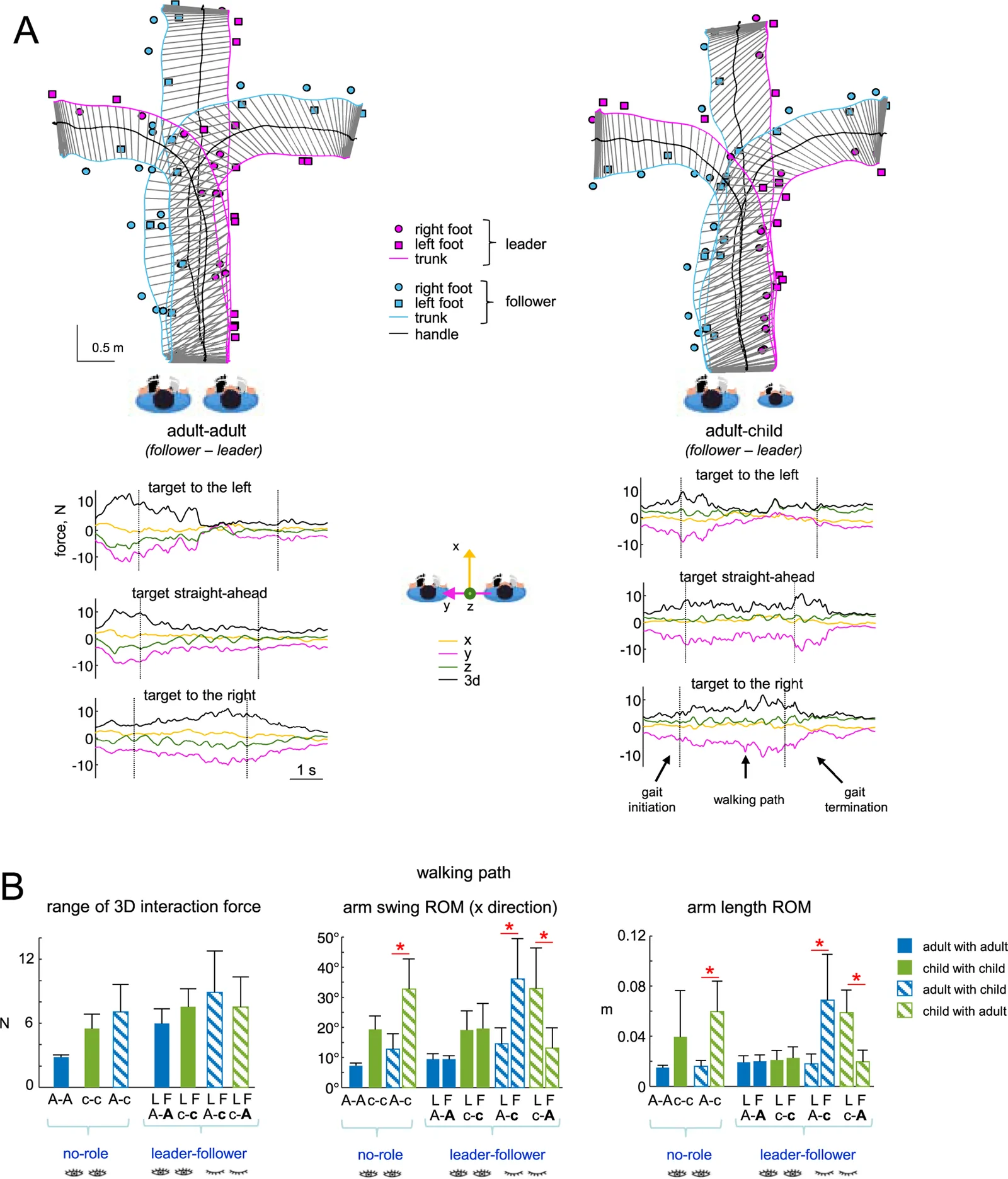
Characteristics of haptic interaction during guided locomotion. **(A)** Examples of walking trajectories (trunk, handle, and foot placements) for two dyads (A–A and A–C) during guided walking toward the left, central and right targets (upper panels). The trunk trajectories of the two partners are schematically joined every 150 m to show the leader’s slightly forward position relative to the follower throughout all trials. The corresponding interaction force components are displayed at the bottom (y represents the line connecting the shoulders of the leader and follower, and x represents the corresponding normal to this line). Gait initiation, walking path and gait termination are separated by the vertical dotted lines. **(B)** Characteristic interaction parameters (mean + SD) during the walking path: range of 3 days interaction forces, arm swing ROM (x direction), and arm length ROM. Horizontal red lines and asterisks denote significant differences between partners of the same dyad (One-way ANOVA with Tukey’s HSD, p < 0.05).

We specifically analysed the walking path, which required one of the partners (blindfolded) to use proprioceptive feedback from the contact arm to follower the leader to different targets (Figure 4). Figure 4A (top panels) illustrates two dyads’ whole-body trajectories to three different targets from a starting position. The trunk trajectories of the two partners show the leader’s slightly forward position relative to the follower throughout all trials. The lower panels display the corresponding interaction forces along x, y, and z, with a different trend of mediolateral force (y) for the right and left targets, as stated and investigated further. Contact forces were generally small (<10 N).

Figure 4B summarises the overall characteristics of interaction forces for all dyads and conditions. In the ‘no role’ condition, oscillations of 3 days forces were relatively smaller in adult (A-A) dyads than in c-c or A-c dyads (Figure 4B, left panel), probably because the range of angular arm oscillations and the range of changes in the arm length were smaller for A-A than in c-c or A-c dyads during hand-by-hand walking (Figure 4B, middle and right panels).

Regarding haptic communication across different age groups, children were more compliant with adults. First, there was an augmented arm swing ROM in the (x) direction of walking in children when they walked hand-by-hand with adults in all conditions (‘no role’, A-c, and c-**A**, Tukey’s HSD, p < 0.05, Figure 4B, middle panel). Second, the range of changes in the arm length (distance between shoulder and 3 MC markers) was also greater in a child walking with an adult (Tukey’s HSD, p < 0.05, Figure 4B, right panel). Similarly, when we estimated the entire arm stiffness as the ratio of the 3 days interaction force range to the arm length range, it was significantly smaller in children (∼180 N/m) compared to adults (∼500 N/m) during walking in adult-child dyads (Tukey’s HSD, p < 0.05). Interestingly, adults displayed reduced upper-limb compliance (higher entire arm stiffness) when guiding children compared to when they guided adults (Tukey’s HSD, p < 0.05). Changes in the mediolateral (y-axis) force were generally greater than those observed in the x- and z-axis force components (Figure 4A). When we correlated changes in the arm length with the mediolateral force changes, in adults, changes in arm length showed a moderate but significant and consistent correlation (r∼0.5) with mediolateral force changes across all conditions (A-A, A-c, A-A, A-c, c-A). In contrast, this correlation was weaker, more variable, and inconsistent in children.

### Directional characteristics of haptic interaction forces in guided goal-directed locomotion

**FIGURE 5:**
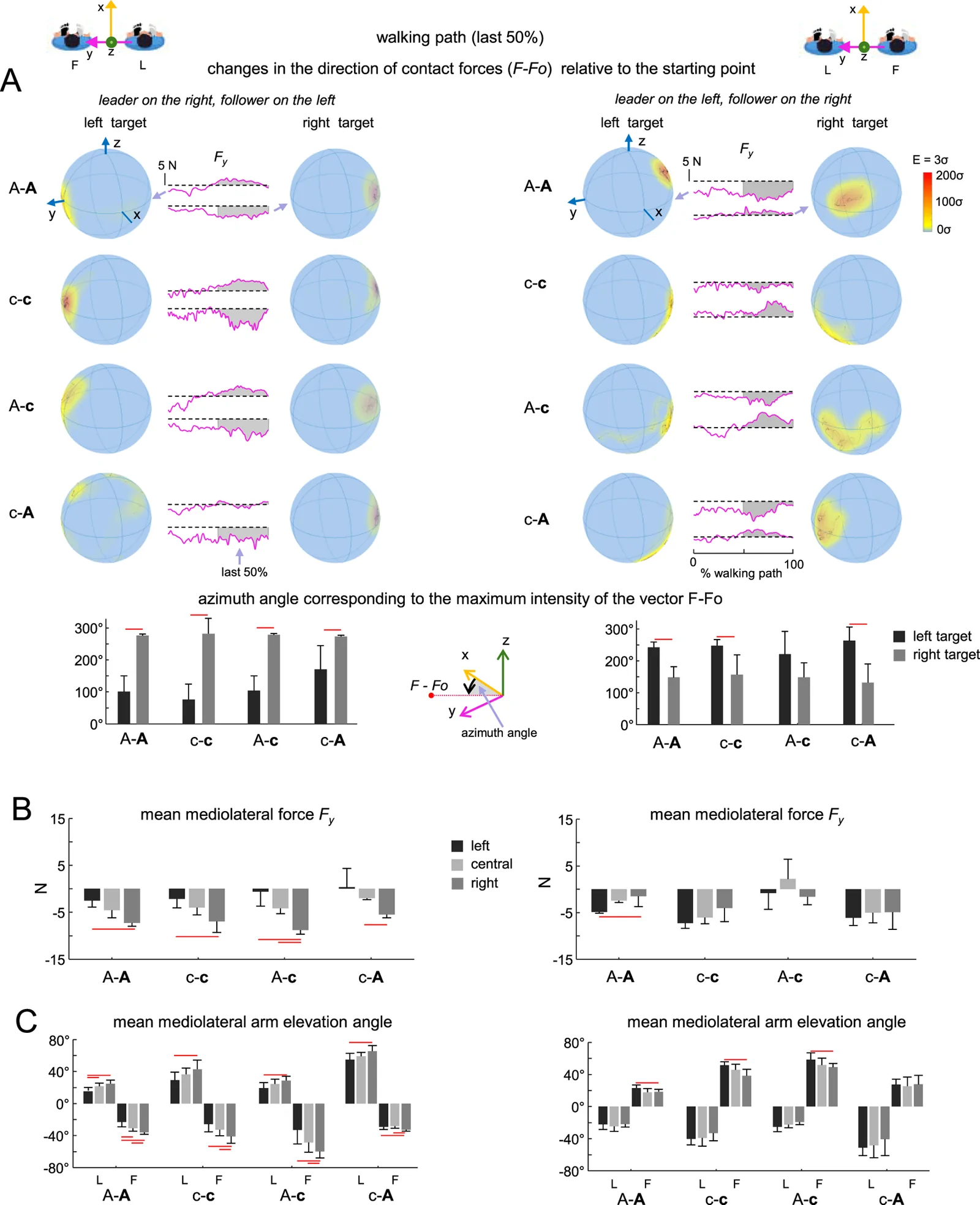
Directional characteristics of contact forces in guided locomotion (during the last half of the walking path). **(A)** Directional characteristics of interaction forces in two configurations: leader on the right, follower on the left (left columns) and leader on the left, follower on the right (right columns). *Top:* spherical spatial density of the force vector directional changes *F* − *F*_*0*_ (i.e., relative to the starting point before gait initiation *F*_*0*_) during the last 50% of the walking path when walking toward left and right targets in representative subjects for each group (A-A, c-c, A-c, and (C–A). Each point corresponds to a single sample (sample frequency 1,000 Hz); the colour scale indicates density diagrams calculated using the Kamb method for directional data with *E* = 3*σ* and exponential smoothing (see Methods). The component *F*_*y*_ of contact force for all dyads is likewise displayed in magenta, along with dotted horizontal lines representing the value at the starting point (*F*_*0*_ *)* prior to gait initiation. Shadow area in grey highlights changes in *F*_*y*_ during the last 50% of the walking path. Note direction-specific differences in *F*_*y*_ when walking towards left vs. right targets in the two configurations. *Bottom*: To evaluate how the orientation of contact forces (*F*) changed during the second half of the walking path compared to their orientation at gait initiation (*F*_*0*_), we computed the spherical distribution of the difference vectors (*F* − *F*_*0*_) (exemplified in top panels) and identified the azimuth angle associated with the region of highest density (mean + SD across trials/dyads). Horizontal red lines denote significant differences (Watson-William test, p < 0.05). **(B)** Mean mediolateral (y direction) forces for the last half of the walking path for the two configurations. **(C)** Mean mediolateral arm elevation angles for the last half of the walking path for the two configurations. Horizontal red lines denote significant differences (One-way ANOVA with Tukey’s HSD, p < 0.05). Note significant differences in the direction of contact forces **(A)**, mediolateral forces **(B)**, and arm elevation angles **(C)** when walking towards different targets.

Finally, we analysed the directional characteristics of haptic interaction forces during guided goal-directed locomotion. Among the trajectories employed, two of them required tuning to the right or to the left, and we compared the characteristics of guided forces for these trials (Figure 5). Because the directional guidance is dependent on whether the leader is on the left or right with regard to the follower, we report the findings from this analysis in two configurations: leader on the right - follower on the left, and leader on the left - follower on the right. Half of the dyads were recorded in the first configuration and the other half in the second, allowing us to compare the two using the spherical density distribution of the force vector (see Methods).

Figure 5 shows the results of this analysis in all dyads for the final 50% of the walking path, where turns are typically made. Figure 5A illustrates examples of spherical spatial density of the force vector directional changes *F* − *F*_*0*_ (i.e., relative to the starting point prior to gait initiation *F*_*0*_) in representative subjects of each group as they walked towards left and right targets. Depending on the turn’s direction, the guided force vectors (*F* − *F*_*0*_) tended to be centred in two distinct y directions. To quantify these differences in force orientation during the last 50% of the walking path, we computed the azimuth angle for the point of maximum intensity. We found that the distribution of forces is primarily shifted to the right or left of the y-axis, depending on the target direction (left or right, p < 0.05, Watson-William test) and the follower’s relative position to the leader (Figure 5A, lower panels). Depending on the spatial arrangement of the two interacting individuals (leader on the left or right), changes in the orientation of 3 days forces during turning towards targets were accompanied by directional changes in the mediolateral force component F_y_ (Figure 5B) and distinct values in the mediolateral arm elevation angle (Figure 5C). It is important to note that, for both adults and children, relatively slight variations in F_y_ (<2–3 N compared to walking straight ahead, Figure 5B) and in mediolateral arm elevation angle (<3–4, Figure 5C) were enough to direct the follower to the left or right. It is also worth noting that in the ‘contact no role’ condition, where both partners moved simultaneously with eyes open, no significant changes were found in the mediolateral F_y_ components and the mediolateral arm elevation angle between turning to the left and right targets (One-way ANOVA, p > 0.05).

### Gait termination

Throughout the trial, the leader guided the walking route with a little forward position in comparison to the follower, and he or she arrived at the goal a little ahead of the follower (Figure 4A). The leader and follower in each dyad reached the targets fairly accurately; through haptic contact, the follower realised that he or she needed to stop during the final gait termination phase and did so in a single stride (Figures 2A, 4A). There was a trend to reduce upper limb activity during this phase, but the effect was not significant probably because the shoulder muscles displayed variable activity or were not very active over the preceding stride and during gait termination.

## Discussion

This study examined guided interactive locomotion in age-diverse pairs (adults and children) walking hand-in-hand, focusing on upper-limb electromyographic activity, limb kinematics, and haptic interaction forces. Handholding naturally occurs between walking partners, providing a developmentally relevant context for investigation. To our knowledge, this is the first study to evaluate interaction forces during side-by-side walking with hand contact in age-mismatched dyads and during guided bidimensional locomotion. We analysed the spatiotemporal features of hand interaction forces across varying trajectories, roles, and age combinations (Figures 3–5). The results are discussed in relation to interlimb coordination and human-human interaction during locomotion with physical contact, revealing key adaptations in follower behaviour and haptic communication. The results are discussed in relation to interlimb coordination and age-related adaptations in human-human physical interaction, highlighting distinct features of follower behaviour and haptic communication across the lifespan.

### Mechanical effect of interactive forces vs. sensory communicative cues in guided locomotion

Applying external forces can mechanically influence upper limb kinematics and alter body trajectory. However, in this study, contact force variations were minimal (2–3 N) yet sufficient to guide movement toward lateral targets (Figure 5B). Given that directional changes occurred in the latter half of the path over ∼2 s (Figure 4A), applying a 3 N mediolateral force (F_y_) to a 30 kg child or a 60 kg adult would yield lateral displacements ( $y=\frac{a\;t^{2}}{2}$ , where *a* = *F*_*y*_ */m* is acceleration, *m* is body mass, and *t* = 2 s) of approximately 0.2 m and 0.1 m, respectively—far less than the observed ∼2 m deviation. These findings suggest that haptic interaction served primarily as a communicative cue rather than producing purely mechanical effects. The frequent activation of the DELTa muscle during gait initiation (Figure 3B) further supports the interpretation of haptic communication in guided locomotion as an active, compliant process rather than a passive one.

The interaction guided forces observed (∼3 N) were comparable to or smaller than those reported in other human-human interaction tasks (Ikeura and Inooka, 1995; Reed and Peshkin, 2008; Wang et al., 2008; Hawkins et al., 2012; Takagi et al., 2019). For instance, forces during partnered forward-backward stepping (Sawers et al., 2017) and for step synchronization during hand-by-hand walking in adults (Sylos-Labini et al., 2018) have been reported in the 5–12 N and 2–5 N range, respectively, supporting the notion that relatively small forces can effectively convey sensorimotor information. Our findings align with this, suggesting that subtle contact forces also serve as communicative cues during guided locomotion.

### Compliant behaviour

Compliant behaviour is as essential as resistive behaviour in everyday motor and postural activities and is often expressed through muscle length changes that elicit involuntary shortening reactions (Andrews et al., 1972; Gurfinkel et al., 1989). It is also noteworthy that compliant behaviour appears to be innate, as shortening reactions are prominently observed in young infants during imposed passive movements (Dolinskaya et al., 2023). This dynamic “postural frame,” inherently embedded in movement and posture coordination, likely underlies both compliant and resistive functional responses (Cacciatore et al., 2014; 2024; Ivanenko and Gurfinkel, 2018) and is particularly relevant for interactive locomotion.

Following a leader inherently involves compliant, rather than resistive, interaction—especially given that followers were explicitly instructed to adapt to the leader’s movements. As noted above, haptic communication during guided locomotion is an active, compliant process rather than a passive one. During gait initiation, we observed a clear manifestation of this compliant behaviour. Specifically, we focused on proximal (shoulder) muscles due to their key role in bipedal gait (La Scaleia et al., 2014), although their activity is typically low and variable, particularly at slower walking speeds when arm swing is reduced (Ivanenko et al., 2006; Kuhtz-Buschbeck and Jing, 2012). No consistent EMG patterns emerged during direction-specific turning, possibly due to this variability (Figure 3A). However, systematic muscle responses were frequently observed in followers during gait initiation (Figure 3), particularly in the anterior deltoid (DELTa), indicating active shortening responses consistent with compliant interaction.

Due to differences in body height between children and adults (1.2 m vs. 1.7 m), the upper limb orientation varied, with children exhibiting greater elbow flexion when paired with adults. Regardless of the exact arm posture, EMG activity in DELTa during gait initiation highlighted compliant follower behaviour. This effect was especially pronounced in asymmetrical dyads (adult-child pairs; Figure 3B), likely due to the increased arm elevation required by the child (Figure 3C), resulting in greater DELTa shortening. While EMG responses showed inter-individual variability, the trend of increased DELTa activation was consistent across dyads (Figures 3B,D), with additional, albeit smaller, increases observed in DELTp and DELTm, possibly reflecting coactivation. The appearance of DELTa EMG activity suggests that followers adopt a compliant motor strategy, characterized by adaptive modulation of muscle responses to the leader’s movements. This compliance likely facilitates physical synchrony and coordination during joint locomotion.

### Haptic communication forces

To quantify interpersonal interaction forces, we analysed the total 3D force, the individual components (x, y, z), and the orientation of the resultant force vector. Interaction forces ranged from approximately 3–8 N, with the smallest magnitudes observed in adult-adult dyads (Figure 4B, left panel). Notably, hand contact during walking substantially reduced arm swing in the contact limb—consistent with prior findings suggesting stabilization of the contact point—while the contralateral arm maintained normal oscillations (Sylos-Labini et al., 2018). In our study, guided arm oscillations were minimal in individual strides (∼5–10), with total ranges of motion (ROM) throughout the walking path between 7 and 35 (Figure 3B, middle panel), smallest in same-height dyads and greatest when a child walked with an adult. This increased motion may reflect biomechanical differences, such as lower limb mass and shorter upper limbs in children, amplifying mechanical oscillations transmitted through the arm.

During hand-by-hand walking, arm length dynamically changes with each stride due to the body’s natural oscillations. These effects were especially pronounced in child-adult dyads, where children exhibited greater oscillations and, consequently, more compliant limb behaviour (Figure 4B, middle and right panels). When we examined the relationship between changes in arm length and mediolateral force changes - which were the most pronounced (Figure 4A) - adults showed a moderate, yet significant and consistent correlation (r∼0.5) across all conditions. In contrast, this correlation in children was weaker, more variable, and inconsistent. This may suggest role- and age-dependent modulation: adults may emphasize precise control and interaction stability, whereas children display more variable and responsive motor behaviour - potentially supporting learning, exploration, or reflecting intrinsically different compliance. The age-related differences observed in our study may reflect developmental variations in sensorimotor and cognitive functioning between younger participants and adults. In children, key mechanisms underlying interpersonal coordination - such as proprioceptive accuracy (King et al., 2010; Cignetti et al., 2017), predictive motor control in the interpersonal coordination (Satta et al., 2017; Meyer and Hunnius, 2020), anticipatory locomotor adjustments (McFadyen et al., 2001; Belmonti et al., 2013; Croix and Korff, 2013), and the ability to integrate haptic cues (Fanghella et al., 2021) - are still maturing. These systems play a critical role in anticipating and adapting to a partner’s movements, and their relative immaturity may lead to less stable or less efficient coordination patterns. Adults, by contrast, typically exhibit fully developed multisensory integration and motor planning capacities, which may facilitate more precise temporal and spatial alignment during haptic interaction. Furthermore, the ability to flexibly shift attention, interpret others’ intentions, and regulate one’s own motor output in response to subtle social cues may be more refined in adulthood, contributing to improved coordination performance. These developmental differences (inconsistent correlation between changes in arm length and mediolateral force changes in children; differences in arm length and arm swing ROM, Figure 4) likely underpin the performance gaps observed across age groups.

We also examined individual force components in the context of guided bidimensional locomotion. The mean lateral (y-axis) force was larger, likely due to arm abduction (Figure 4A), while oscillation amplitudes were comparable across all axes. The mediolateral (y) component consistently varied during turns (Figures 4A, 5A), remaining small during straight walking (2–5 N), and requiring only minor adjustments (2–3 N) for target-directed turning (Figure 5B).

Corresponding analyses of force orientation revealed systematic directional shifts in interaction vectors across target directions (Figure 5). Spherical density plots indicated forces were primarily oriented along the y-axis - toward the negative y-axis for rightward targets and the positive y-axis for leftward ones (Figure 5A). Significant azimuth angle differences between these conditions highlight the role of haptic cues in refining locomotor trajectory. These findings suggest that haptic guidance adapts to intended direction, offering the follower a meaningful sensory signal for spatial adjustment during joint movement.

## Limitations

This study has several limitations. First, the relatively small sample size (11 dyads) may reduce the generalizability of the findings and limits statistical power, particularly when examining subtle age- or role-dependent differences. Second, the age range of the participants was restricted to children aged 6–8 years and adults, excluding other developmental stages such as adolescence or older adulthood. This narrows our understanding of how haptic communication evolves across the lifespan. This choice was deliberate, as our aim was to compare a clearly defined developmental stage in childhood with the mature stage of adulthood, in order to highlight potential differences in haptic communication at two distinct points of the lifespan. Third, only female adults were included, potentially introducing gender bias. The inclusion of male participants could reveal important sex-related differences in motor strategies and interpersonal coordination. Fourth, the cross-sectional design limits insights into how haptic communication and compliant behaviour develop within individuals over time. A longitudinal approach would be necessary to assess the developmental trajectory of these interactive processes. Addressing these limitations in future work would enhance our understanding of the developmental and contextual dynamics of haptic-guided locomotion. Nevertheless, although the sample size is relatively small, the results were consistent across individuals and clearly showed several key age-related features. Also, this study was designed as a preliminary investigation with healthy individuals to explore the feasibility and reliability of the proposed experimental protocol before applying it to clinical populations. Moreover, the controlled laboratory setting may not fully represent naturalistic locomotor interactions in daily life. Future studies with larger, more diverse cohorts, longitudinal designs, and ecological settings are needed to address these limitations and deepen understanding of interpersonal motor coordination across the lifespan.

### Future directions and applications

The findings have practical implications for gait rehabilitation, developmental studies, and the design of assistive technologies. These interactions can reveal impairments in sensorimotor control and offer insights into altered neural processes. For example, upper limb compliance, which reflects dynamic muscle tone (Bernstein, 1940), plays a key role in locomotor control and is particularly important during interactive walking tasks, especially when haptic interaction with objects and people has a critical role in the development of locomotion at an early age (Karasik et al., 2011; Heiman et al., 2019). Impaired muscle tone may compromise quadrupedal coordination, and haptic interactions may serve as valuable tools to assess the influence of such impairments on gait (Ivanenko et al., 2013; Cacciatore et al., 2024).

Clinical populations with disrupted sensorimotor integration, such as children with cerebral palsy, individuals with diabetic neuropathy, stroke, cerebellar ataxia, could benefit from haptic training protocols. Prior studies have shown that even minimal upper limb support, such as light contact with an anchored railing during treadmill walking, can improve stability and walking outcomes in both healthy individuals and patients (Dickstein and Laufer, 2004; Oates et al., 2017). Similarly, guided walking tasks involving patient-clinician handholding, collaborative object transport, or patient-robot interaction can provide not only postural support but also meaningful sensory cues that promote locomotor adaptation during overground walking. Moreover, interactive locomotion involving handholding often induces spontaneous step synchronization - an effect that could be leveraged to improve spatiotemporal gait features without explicit cues. Since forced synchronization has been shown to be less effective in adapting asymmetrical gait patterns (Nessler et al., 2015), using non-verbal haptic communication may offer more flexible and personalized rehabilitation strategies. Finally, recent work suggests that patient-robot haptic interactions could also be beneficial for training in human-robot collaborative tasks and collective transport, improving step timing, and facilitating collaborative movement goals (Wu and Ting, 2025). Together, these insights underscore the value of upper limb haptic interactions as a promising framework for enhancing both the assessment and recovery of locomotor function across diverse clinical populations, including developmental contexts where age-related motor characteristics must be considered.

## Conclusion

This study demonstrates that hand-in-hand guided locomotion between individuals of different ages - particularly between adults and children - relies on subtle, compliant motor responses and low-magnitude interaction forces to support effective interpersonal coordination. Our findings suggest that these interaction forces (typically around 3 N) function primarily as communicative cues rather than as direct mechanical drivers of movement. The emergence of systematic EMG responses, particularly during gait initiation, underscores the active role of the follower in adapting to the leader’s motion through compliant upper limb behaviour. This was especially evident in asymmetrical dyads, such as adult-child pairs, where biomechanical and kinematic adaptations amplified these effects. These adaptations likely reflect developmental differences in motor control and postural tone, as well as differences in body size.

Together, these results advance our understanding of how physical contact during locomotion facilitates coordination via compliant interaction and haptic communication. They provide a foundation for future studies on interpersonal motor behaviour, as well as potential applications in physical therapy (both for diagnostic purposes, e.g., assessing dynamic postural tone, spasticity or haptic communication abilities, and for gait rehabilitation), developmental assessment, robotics and assistive technologies involving human guidance or support during locomotion, where age-related motor characteristics must be considered.

## Data Availability

The original contributions presented in the study are included in the article/Supplementary Material, further inquiries can be directed to the corresponding authors.
