# Correction: Haptic communication and interpersonal dynamics in hand-by-hand guided locomotion in children and adults

**DOI:** 10.3389/fbioe.2026.2035622

**Published:** 2026-10-30

**Authors:** Priscilla Avaltroni, Francesca Sylos-Labini, Margherita Villani, Germana Cappellini, Francesco Lacquaniti, Yury Ivanenko

**Affiliations:** 1 Laboratory of Neuromotor Physiology, Istituto di Ricovero e Cura a Carattere Scientifico Fondazione Santa Lucia, Rome, Italy; 2 Department of Systems Medicine and Center of Space Biomedicine, University of Rome Tor Vergata, Rome, Italy

**Keywords:** two-dimensional interactive locomotion, children, haptic interaction forces, EMG activity, interpersonal coordination, human gait

An incorrect **Funding** statement was provided (the specific grant code CUP J83C22002060007 was omitted). The correct funding statement reads:

“This work was supported by the Italian Ministry of Health and funded by the European Union - Next-Generation EU - NRRPM6C2 - Investment 2.1 Enhancement and strengthening of biomedical research in the NHS, PNRR-POC-2022-12375705, CUP J83C22002060007”.

The original version of this article has been updated.

